# Estimating the production of withaferin A and withanolide A in *Withania somnifera* (L.) dunal using aquaponics for sustainable development in hill agriculture

**DOI:** 10.3389/fpls.2023.1215592

**Published:** 2023-08-31

**Authors:** Manali Singh, Shivani Bhutani, Nisha Dinkar, Anita Mishra, Kahkashan Perveen, Alanoud T. Alfagham, Mehrun Nisha Khanam, Santosh Chandra Bhatt, Deep Chandra Suyal

**Affiliations:** ^1^ Department of Life Sciences, Parul Institute of Applied Sciences, Parul University, Vadodara, India; ^2^ Department of Biotechnology, Invertis University, Bareilly, India; ^3^ Department of Biotechnology, Khandelwal College of Management Science and Technology, Bareilly, India; ^4^ Department of Science, Vidyadayini Institute of Science, Management and Technology, Bhopal, India; ^5^ Department of Botany & Microbiology, College of Science, King Saud University, Riyadh, Saudi Arabia; ^6^ Research Centre for Plant Plasticity, School of Biological Sciences, Seoul National University, Seoul, Republic of Korea; ^7^ Kumaun University, Nainital, Uttarakhand, India

**Keywords:** aquaponics, withania somnifera, metabolites, withaferin A, withanolide

## Abstract

**Introduction:**

Humanity is suffering from huge and severe difficulties, including changes in climate, soil degradation, scarcity of water and the security of food and medicines, among others. The aquaponics system acts as a closed loop consisting of aquaculture elements and hydroponics, which may contribute to addressing these problems. The aquaponics method is quickly expanding as the requirement to increase the production of sustainable herbal products, including medicinal compounds and foods, in freshwater systems and replenish phosphorous reserves shrinks.

**Methods:**

The current work is designed to increase the production of the antioxidants withaferin A and withanolide A in two varieties (Jawahar-20 and Poshita) of *W. somnifera* using the aquaponics technique. Total 100 seedlings (one month old) grown in soil initially were taken to be grown in aquaponics for a time period of 6 months.And 100 seedlings were placed in pots containing soil as control for study after six months.

**Results:**

It was observed that the higher content of withaferin A was analyzed in the root and stem samples of Jawahar-20 and Poshita from the six-month-old plant of *W. somnifera*. The maximum content of withanolide A was examined in the root samples of the six month-old plants of Poshita (1.879 mg/g) and Jawahar-20 (1.221 mg/g). While the 6 month old Poshita seedling grown in soil recorded less withaferin A (0.115 ± 0.009^b^) and withanolide A (0.138 ± 0.008^d^).

**Discussion:**

It is concluded that Poshita was found to be more promising for the enhanced production of withaferin A and withanolide A in the aquaponics system. Moreover, the root was observed as the best source for the production of withaferin A and withanolide A and the best age of the plant is 2 years for the production compounds in medicinal plants with futuristic perspective to hill agriculture integrated farming. compounds. Thus aquaponics can be an effective approach with enhanced yield of bioactive compounds in medicinal plants with futuristic perspective to hill agriculture and integrated farming.

## Introduction

Farmers in the hilly regions face a major constraint due to undulating topography, leading to soil erosion gradually causing loss of its fertility. Thus most of the farming is affected and is performed on fragile land set-ups, then lack of infrastructure, lack of transport, irrigational problems, lack of capital are another huge challenges faced by farmers of hilly regions. This affects the growth and production of quality crops inspite of hard efforts and funds of the farmer. Moreover the exposure of crops to different types of biotic and abiotic stress make the crops more vulnerable affecting its yield. Thus in order to sustain such harsh conditions integrated farming approach offers a great advantage to the farmers by utilization of available resources in a precise manner to get the best results. Aquaculture offers a promising approach to cater to the needs of farmer and also enable him to raise his income. Aquaculture comprises three entities: fish, plants, and nitrifying bacteria. Where by the nitrifying bacteria convert the unfed food and faecal waste of the fish into utilisable simpler forms, i.e., converting ammonia into nitrates that can be taken up by the hydroponic plants. Thus, aquaponics serves as a source of nutrition for the hydroponic plants under controlled conditions. Aquaponics is resilient integrated farming with less water, labour, and land, providing better nutrition and wellness to the growing plants and crops. Different plants grow successfully in aquaponic systems. Fish rearing and hydroponic vegetable components must be operated continuously in order to have enhanced production (Rakocy et al., 2007). Hydroponically grown plants require a lower pH, from 5.5 to 6.5 (Rakocy et al., 2007). The maintenance of nutrient solution pH is a significant challenge in aquaponics systems, as not only it promotes plant growth but also influences the bioactive metabolite production, root rhizosphere and apoplastic pH. In a study on Taraxacum officinale and Reichardia picroides, low pH levels (pH = 4.0) seemed to be beneficial to nutritional and dietary value in both species highlighting the potential of commercial cultivation under adverse conditions, especially in sustainable farming systems (Alexopoulos et al., 2021). Aquaponics is a symbiotic combination of growing fish and hydroponics, where nutrient-enriched water is used for growing plants in a soil-less culture. Thus, aquafarming is an integrated farming method for growing plants under controlled environmental conditions. It can be a powerful method for the development of elite germplasm with enhanced pharmaceutically active ingredients from medicinal plants in hilly and desert areas where land and water are scarce. Traditional practices of uprooting the whole plant for the extraction of plant metabolites can lead to the extinction of many important plant varieties of pharmaceutical significance. Thus, conservation of such endangered medicinal plant species is the prime need for adopting sustainable agricultural and integrated farming methods.

A hydroponic-centered system for the production of crops provided the necessary nutrients to produce cherry tomatoes (Schmautz et al., 2016) and reported microbial niche distinctions within the aquaponics system (Schmautz et al., 2017). The aquaponics system is also regulated by the type of fish being used for the maintenance of the aquaponics system. Generally, the species used for aquaponics production include Arctic char, trout, perch (Diver and Rinehart, 2010), bluegill, largemouth bass, channel catfish, barramundi, Murray cod, jade perch (Nelson and Pade, 2008), koicarp, goldfish, pacu, and common carp (Rakocy et al., 2007).

Medicinal plants have a very significant role in curing different ailments naturally (Bhasin et al., 2019). The pharmacologically significant medicinal plants are in huge commercial demand owing to the presence of bioactive ingredients (Singh et al., 2017; Gupta et al., 2019). The bioactive metabolites of *Withania somnifera* possess adaptogenic, anticancer, anti-convulsant, immunomodulatory, antioxidative and neurological effects. The plant has also been found to be efficient in the treatment of osteo-arthritis, geriatric, behavioural and anxiety (Singh et al., 2022). The root extract of Ashwagandha containing withanolide A is reported to have health promoting effects such as anti-stress, anti-arthiritic, anti-inflammatory, analgesic, anti-pyretic, anti-oxidant and immunomodulatory properties (Singh et al., 2022). Withanolide B, D, F and withanosides (glycosylated steroids) secreted by WS having neuroprotective, anticancer, hepatoprotective, anti-aging, diuretic, antipogenic, hemopoietic, immunomodulatory functions and antioxidant activities (Singh et al., 2017). It is also reported to improve the overall health so works as a rejuvenating drug to the aged persons. However roots of the plant is used in preparing tonic which promote anti-aging properties and also helpful in the treatment of infectious diseases.

The major bottleneck is the availability of raw materials to meet commercial demands (Singh et al., 2018). It has been reported that the contents of bioactive compounds vary from tissue to tissue and variety to variety, so the selection of elite plant varieties is crucial (Singh et al., 2019). The bioactive compounds can be enhanced by the application of different elicitors, in different environmental regimes, and through tissue culture techniques (Singh et al., 2020). It has been reported that 15 mg/L ammonium nitrate increased the withanolide contents (1.74 mg/g DW withanolide A, 0.92 mg/g DW withanolide B, 0.52 mg/g DW withaferin A, and 1.54 mg/g DW withanone) in a culture of roots formed from shoot regeneration (Sivanandhan et al., 2015).

In aquaponics systems, ammonia is an important nitrogen source for plants, and fish species that accumulate ammonia and urea in higher quantities are mostly in demand. As a result of the stability of the nitrogen metabolism processes, tolerant temperature species are preferred for higher urea and ammonia excretion ratios. The main obstacle to the aquaculture technique is represented by its first drawback: high initial capital investments to maintain a constant water quality that will respond to the physiological requirements of cultured species. People have also reported that excess solid wastes increase the BOD, causing a lowering of oxygen levels in the rhizosphere and the accumulation of ammonia and nitrate, which are toxic for plant growth (Rakocy et al., 2012; Danaher et al., 2013). Pharmaceutically active metabolites of *Withania somnifera* Withanolide A and Withaferin A are of immense health benefits to humans.The studies have shown that withanolide A possess neuroprotective, anticancer, hepatoprotective, anti-aging, diuretic, antipogenic, hemopoietic, immunomodulatory functions and antioxidant activities, possess strong neuropharmacological efficacy (Singh et al., 2022). Withaferin A serves as a potent anti-cancerous compound. It also has diverse pharmacological activities, including antitumor, antiangiogenic, cardioprotective, anti-inflammatory, and immunomodulatory effects. Recently it was observed that Withaferin A having therapeutic potential, protects from COVID-19 infection to mitigate the virus made cardiovascular disease (Singh et al., 2022).The two predominant phytochemicals of the plant contribute in drugs as well as assists with getting the physiological property for the treatment of various illnesses.

Keeping in mind the significance of aquaponics, the present study focuses on an aquaponics system that has been employed for the enhancement of withanolide contents under the controlled micro and macro environments of rhizogenesis.

## Materials and methods

### Material prepared or purchased

The seeds of commercially available varieties of *Withania somnifera* (L.), viz. Jawahar-20 and Poshita, were purchased from CIMAP, Lucknow. The fish and their meals were purchased from the certified center of the G. B. Pant University of Agriculture and Technology, Pantnagar.

### Aquaponic culture for Poshita and Jawahar-20 varieties of *Withania somnifera*

The study was performed to analyze the production of antioxidants withaferin A and withanolide A in two elite varieties, Jawahar-20 and Poshita, of *W. somnifera* Dunal using modern hydro-chemical aquaponics culture techniques. Total 100 seedlings which one month old grown in soil initially were taken to be later grown in aquaponics for 6 months and 100 seedlings were placed in pots containing soil as control for study after six months. The aquaponics culture condition was maintained between 65-85°F, and the pH between 5.5 to 6.5 was maintained and monitored at every hour interval. The fishes used in aquaculture were Rohu (*Labeo rohita*), Glass Catfish (*Kryptopterus bicirrhis*), Basa (*Pangasius bocourti*), and Singhi (*Heteropneustes fossilis*) fishes were used in the aquaponics system. The feed for the fish was wheat flour (Atta), wheat bran (Choker), azolla (*Azolla caroliniana*), linseed meal, and rice husk powder. The element composition of water used was rich in NH_3_ and other important minerals (**Table 1**). The cultivation time of studied plant of aquaponics was 1 month (control) and 6 (month) seedlings inorder to assess the withanolides production.

**Table 1:** Element analysis of the water used in aquaponic system.

S. No.	Parameters	Tap water	Aquaponic water
1.	pH	7.7	6.5
2.	EC	251 µS/cm	205 µS/cm
3.	Temperature	25°C	28.66°C
4.	Dissolved O_2_	4.1 mg/l	6.81 mg/l
5.	TS	281 mg/l	298 mg/l
6.	TDS	161 mg/l	150 mg/l
7.	BOD	5.95 mg/l	2.95 mg/l
8.	COD	6.38 mg/l	11.71 mg/l
9.	Salinity	12	21
10.	Nitrite(NO_2_)	0.009 mg/l	0.018 mg/l
11.	NH_3_	0.253 mg/l	0.590 mg/l
12.	PO_4_	0.95 mg/l	0.06 mg/l
13.	Cd	0.112 μg/ml	0.034 μg/ml
14.	Zn	0.086 μg/ml	0.035 μg/ml
15.	Cu	0.037 μg/ml	0.308 μg/ml
16.	Cr	0.00	0.00
17.	Pb	0.00	0.297 μg/ml
18.	Na	4.68 ppm	15 ppm
19.	K	0.95 ppm	08 ppm
20.	TDS	161 mg/l	309 mg/l
21.	Mn	0.097 mg/l	0.84 mg/l
22.	Ca	3.205 μg/ml	1.19 mg/l
23.	Mg	14.76 μg/ml	11.02 μg/ml
24.	Hg	7.376 μg/ml	6.02 μg/ml
25.	Fe	2.373 μg/ml	3.56 μg/ml

### Comparison of *Withania somnifera* seedlings grow in soil and aquaponics

The seedlings of the Jawahar-20 and Poshita varieties of *W. somnifera* were also grown in soil. The comparative studies were performed on seedling growth in soil and aquaponics techniques.

### Estimate the content of withaferin A and withanolide A in the leaves, roots and stem samples of Poshita and Jawahar-20 varieties of *Withania somnifera*

The samples of fresh leaves, stems, and roots were taken from the one- and six-month-old seedlings of Jawahar-20 and Poshita varieties of *W. somnifera*. The tissue samples were subjected to drying in a hot air oven at 40°C for 3–4 days until a constant dry weight was obtained. Then the plant tissue was ground with the help of a clean and dry mortar and pestle. Dry powder (1g) of plant tissue was taken and percolated in 50 ml of 80% methanol, then sonicated for 20 minutes and placed on a rotatory shaker at 30°C at 100 rpm overnight. The procedure was repeated three times, and the methanolic extracts thus obtained after percolation were pooled together and filtered through Whatman filter paper (pore size 11µm). Then the methanolic extract was subjected to drying using a rotatory vacuum evaporator maintained at 60°C until a completely dried residue was obtained. The dried residue was dissolved in HPLC-grade methanol (4 ml). A pinch of charcoal was added to the extract in order to decolorize the sample, which was centrifuged at 8000 rpm for 15 min. The supernatant was then filtered through nylon filter membranes (0.22). The samples were used for the estimation of the content of withaferin A and withanolide A. The extra samples were kept in the vials at 4°C for future uses (Singh et al., 2018; Singh et al., 2020). The quantification of antioxidants was done through HPLC in one-month-old seedlings and six-month-old seedlings of Jawahar-20 and Poshita varieties of *W. somnifera* (Singh et al., 2018).

### Essential elements and other parameters analysis in the water sample of aquaponics culture

The analysis of elements existing in the aquaponics water sample used for the elicitation experiment was done through the protocol of *Standard Methods for the Examination of Water and Wastewater* by the *American Public Health Association* (APHA) with a few modifications (Lipps et al., 2023).

### Atomic absorption spectrophotometer

The presence of cadmium, zinc, copper, chromium, and lead was determined by AAS (Atomic Absorption Spectrophotometer (AAS), Benchtop Thermo Fisher Ice 3500 Atomic Absorption Spectrometer. Digestion of the sample (50 ml) with HNO_3_ (20 ml) then the volume of the sample was reduced on a hot plate to 15 ml at 100°C. The final volume was made up to 100 ml with double-distilled water. The sample was then filtered with Whatman filter paper, and the reading of the sample was taken through AAS.

### Spectrophotometric analysis

The presence of Nitrite (NO_2_^-^), Ammonia (NH_3_^+^), and Phosphate (PO_4_^-^) was done through a spectrophotometer, Evolution 201 UV-Vis spectrophotometer (Thermo Scientific, USA),. The wavelength used was 190 nm for Nitrite, Ammonia and Phosphate estimation.

### Flame photometer

Five ml of the sample was taken and filtered with Whatman filter paper for the analysis of the presence of sodium (Na) and potassium (K). The equipment (Microprocessor Flame Photometer, LABTRONICS Model Name/Number: LT-6710) was calibrated with the standard solution of Na and K with a capillary tube (10, 20, 30, 40, 50, 60, 70, 80, 90, and 100 ppm). The sample was put into the equipment to take readings after calibration.

### Biochemical oxygen demand

BOD is the measurement of the amount of dissolved oxygen needed by aerobic biological organisms to break down the organic material present in a given water sample at a certain temperature over a certain time period.

### Procedure to determine the biochemical oxygen demand of water

To determine BOD, two bottles (300 ml) were filled with 10 ml of sample, and the remaining volume was made up with water. The other two BOD bottles were only filled with water, which served as a control. The bottles were closed immediately to avoid any air bubbles in the bottles. Then the bottles were incubated at 20°C for 5 days. After 5 days, the BOD of the sample was analyzed.

### Neutralization of sample

A sample of 50 ml was taken in a 100-ml beaker. The pH of the solution was kept at 7.0 by using 1N H_2_SO_4_ or 1N NaOH. The volume of H_2_SO_4_ or NaOH used to adjust the pH of the 50-ml sample to 7.00 was recorded. Then the volume of sulfuric acid or sodium hydroxide required to neutralise the 1000-ml sample was calculated.

### Removal of chlorine content

The removal of chlorine from the water sample was done by adding sodium sulfite to the sample. A 50-ml water sample was taken, to which 2.5 ml of acetic acid (50%) was added, followed by 2.5 ml of a 10% w/v solution of potassium iodide. After some time, 1 ml of starch indicator was added and titrated with a 0.025 N sodium sulfite solution.

### Preparation of alkali-Iodide-azide reagent

An amount of 500 g of sodium hydroxide (NaOH) and 135 g of sodium iodide (NaI) were dissolved in distilled water, and the final volume was made up of 1000 ml of distilled water. To which 10 g of sodium azide were added.

### Preparation of aqueous solvent

Five liters of double-distilled water were taken in a glass container and aerated with clean compressed air for 12 hours. It was then allowed to get stable for at least 6 hours at 20°C. After that, 5 ml of a 27.5% (w/v) solution of calcium carbonate, 5 ml of a 22.5% (w/v) solution of magnesium sulphate, 5 ml of a 0.15% (w/v) solution of ferric chloride, and 5 ml of a phosphate buffer solution were added. The solution was mixed well and allowed to stand for 2 hours.

### Chemical oxygen demand

Standards were prepared using KHP (potassium hydrogen phthalate). Water sample (2 ml) was added to each vial. In the case of the “blank,” 2 ml of double distilled water was added. Then 2 ml of the standard was added to the corresponding vials. Each vial was mixed well and placed into the COD reactor block for two hours. After two hours, the vials were removed from the block to a cooling rack for about 15 minutes. The readings were taken with the help of a colorimeter.

### Analysis of essential elements and other parameters in the soil sample

Various essential elements and other parameters were analyzed in the soil samples of the mango garden, the transgenic laboratory, and the control soil sample. The soil (vermin-compost added and autoclaved) used for potting the plants of *W. somnifera* in the mango garden, Dept. of Plant Physiology, G.B. Pant University of Agriculture and Technology, and the other in the controlled environment of the Transgenic Laboratory, Dept. of Molecular Biology and Genetic Engineering, G.B. Pant University of Agriculture and Technology, was done through DTPA (diethylene triamine pentaacetic acid).

### Estimation of micronutrients in soil by extraction method

Ten gram of air-dried soil was weighed in a 150-ml conical flask to which 20 ml of DTPA extraction buffer was added (1.967 g DTPA, 14.9 g TEA (triethanolamine), and 1.47 g CaCl_2_.2H_2_O were dissolved in 200 ml DW). The pH of the solution was adjusted to 7.0, and the final volume was made up to 1000 ml. The flask was tightly capped with a polyethylene stopper and then kept on a horizontal shaker (120 cycles/min) for 2 h. After shaking, the filtrate was filtered through Whatman filter paper No. 42, and the filtrate was used for the estimation of micronutrients. The calculation of the extractable micronutrient content was done with the help of the following equation:


DTPA extractable micro−nutrient content(mg/kg soil)=filtrate concentration(μg/ml)×20/10

## Results and discussion

### Aquaponic culture for Poshita and Jawahar-20 varieties of *Withania somnifera*

**Figure 1:**
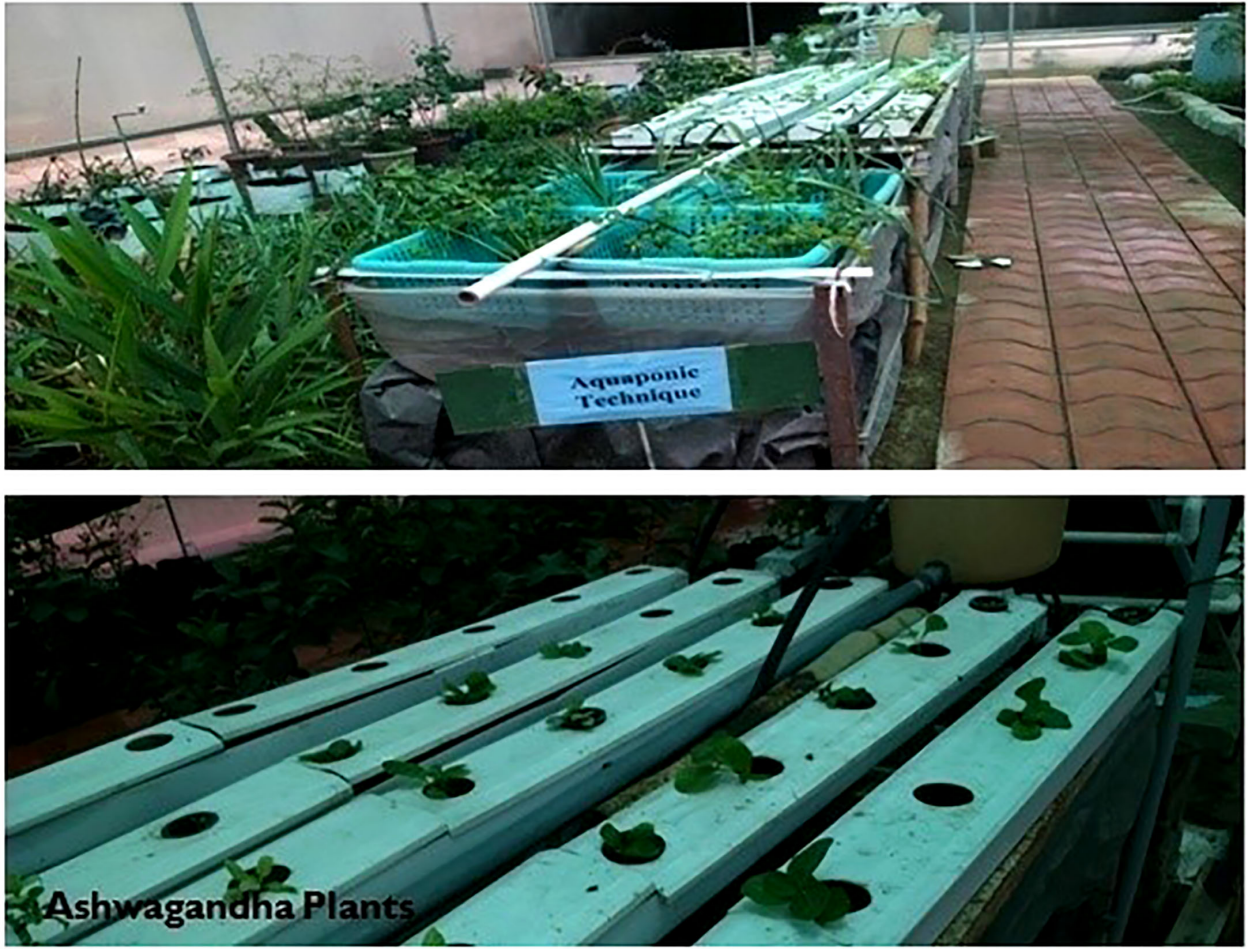
Seedlings of *Withania somnifera* in the control condition of aquaponics.

The seedlings of the two promising varieties, Jawahar-20 and Poshita of *W. somnifera*, were grown in controlled conditions using the hydro-chemical technique of aquaponics for the estimation of biomass and content of withaferin A and withanolide A (**Figure 1**).

### Comparison of seedlings of *Withania somnifera* grown in soil and aquaponics

**Figure 2:**
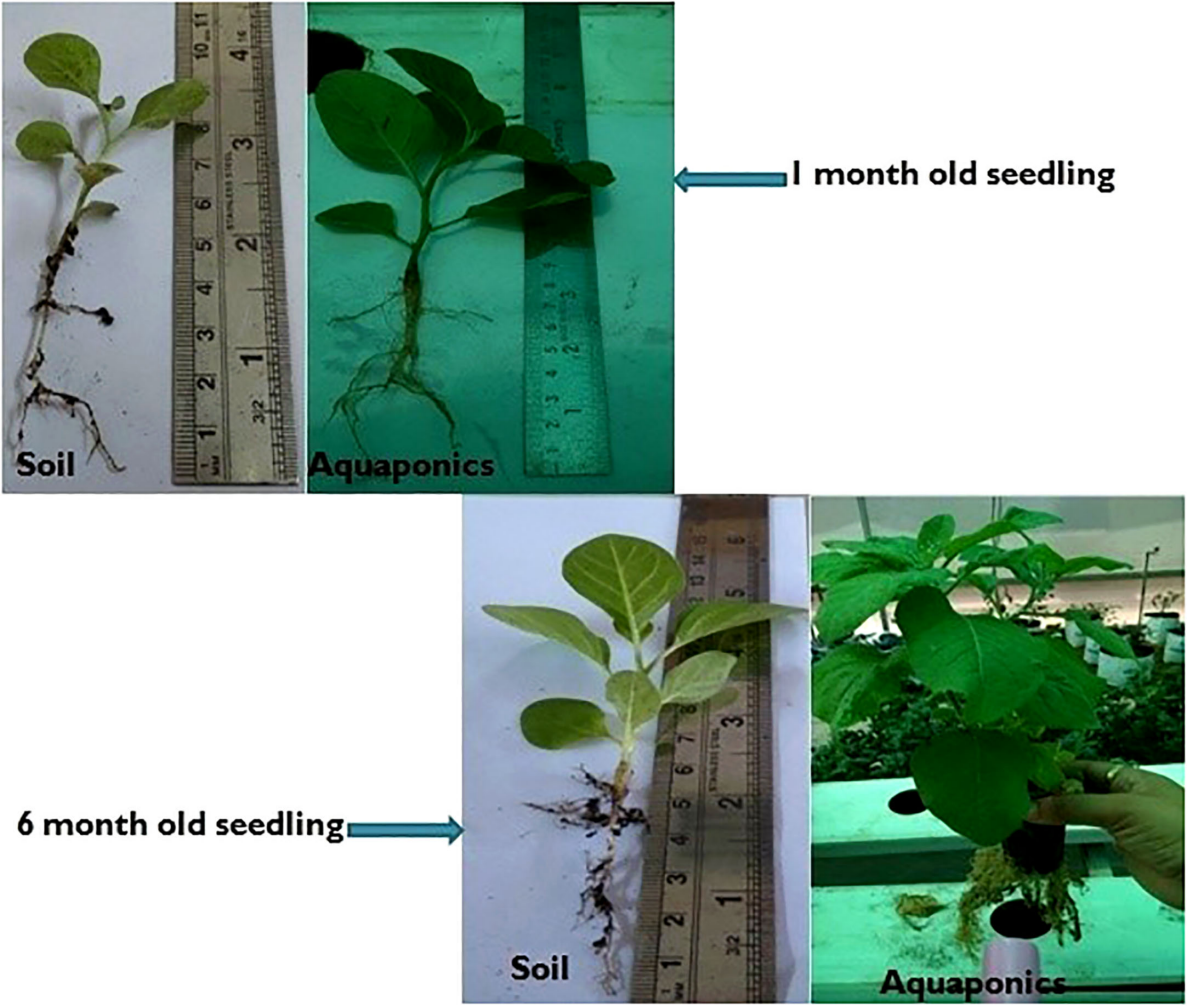
Comparison of seedlings of *Withania somnifera* grown in (i) Soil (ii) Aquaponics.

The comparative studies showed more growth in the seedling of the one-month-old *W. somnifera* plant in aquaponics culture in comparison to soil culture. Similarly, the results demonstrated more length and biomass in aquaponics culture in six-month-old *W. somnifera* plants (**Figure 2**). The growth of seedlings in the aquaponics system was also positively controlled by different species of fish (Knaus and Palm, 2017). The fish used in the present study of the aquaponics system were Rohu (*Labeo rohita*), glass catfish (*Kryptopterus bicirrhis*), Basa (*Pangasius bocourti)*, and Singhi (*Heteropneustes fossilis*). Previous studies have investigated the importance of African catfish (*Clarias gariepinus*) in aquaponics systems (Endut et al., 2009; Palm et al., 2014). The different types of feed given to the fish in our aquaponic system were also helpful for the regulation of the growth of the plant *W. somnifera*. One study has demonstrated that the fish feed rate is associated with the growth of plants, but the conversion of feed and nutrient assimilation vary with feed type and plant crop type (Rakocy et al., 2007).

### Estimation of Withaferin A and Withanolide A in different plants tissues of Ashwagandha

**Figure 3:**
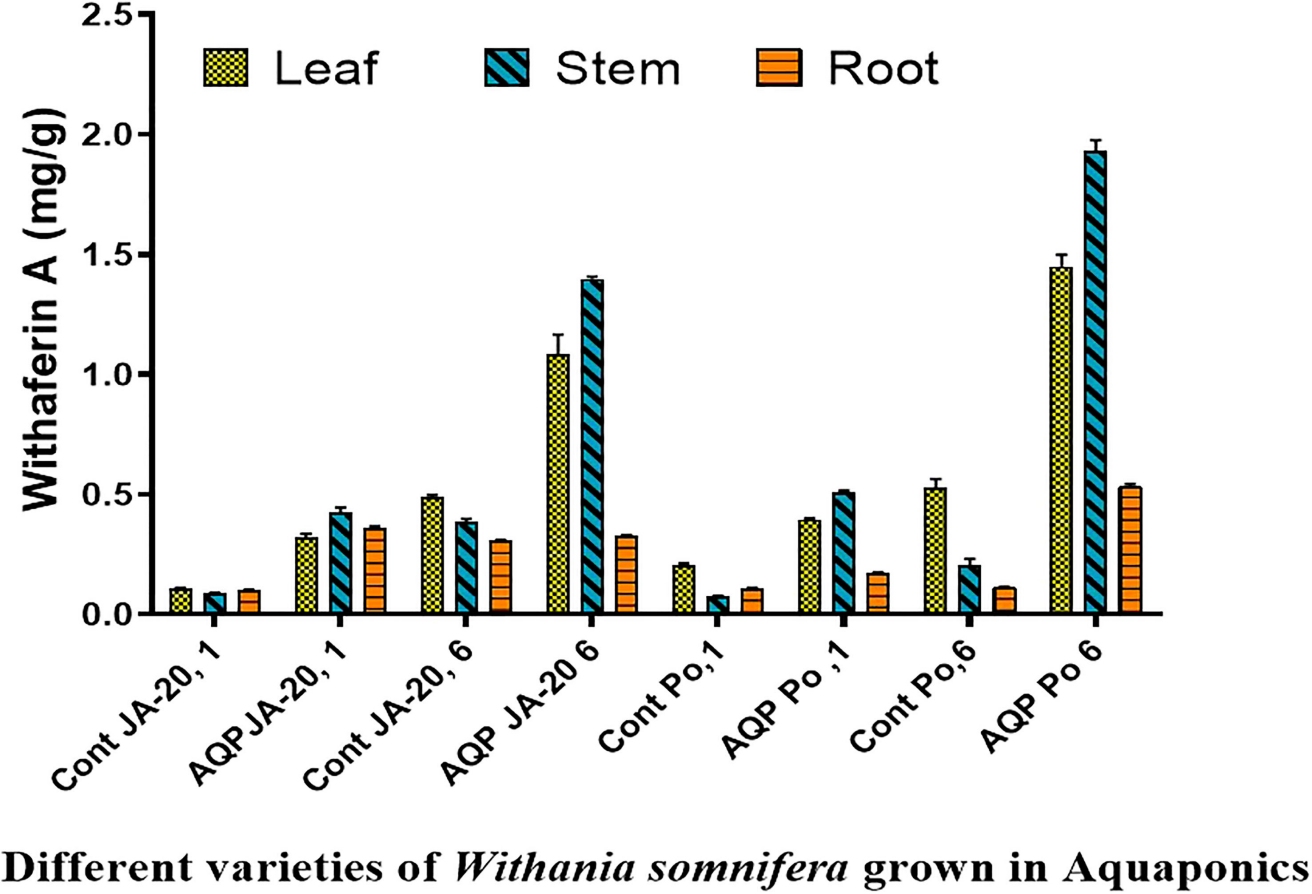
Withaferin A, content in the two varieties of *Withania somnifera* (Jawahar-20 and Poshita) grown in aquaponics (where Cont JA-20, 1 = 1 month Jawahar seedling grown in soil; AQP JA-20, 1 = 1 month Jawahar seedling grown in aquaponics; Cont JA-20,6 = 6 month Jawahar seedling grown in soil; AQP JA-20,6 = 6 month Jawahar seedling grown in aquaponics; Cont PO1 = 1 month Poshita seedling grown in soil; AQP PO1 = 1 month Poshita seedling grown in aquaponics; Cont PO,6 = 6 month Poshita seedling grown in soil; AQP PO,6 = 6 month Poshita seedling grown in aquaponics.

The higher content of withaferin A was examined in the stem (0.446 mg/g), followed by the root (0.367 mg/g), and leaves (0.336 mg/g) of Jawahar-20 in one-month-old plants, while the higher content of withanolide A was examined in the root (0.149 mg/g), followed by stem (0.143 mg/g), and leaves. The one-month-old plants of Poshita showed a higher content of withaferin A in the stem (0.516 mg/g), followed by the leaves (0.400 mg/g), and the root (0.175 mg/g). The one-month-old plants of Poshita showed a higher content of withanolide A in the root (0.252 mg/g), followed by the leaves (0.168 mg/g), and the stem (0.066 mg/g) (**Figure 3**).

**Figure 4:**
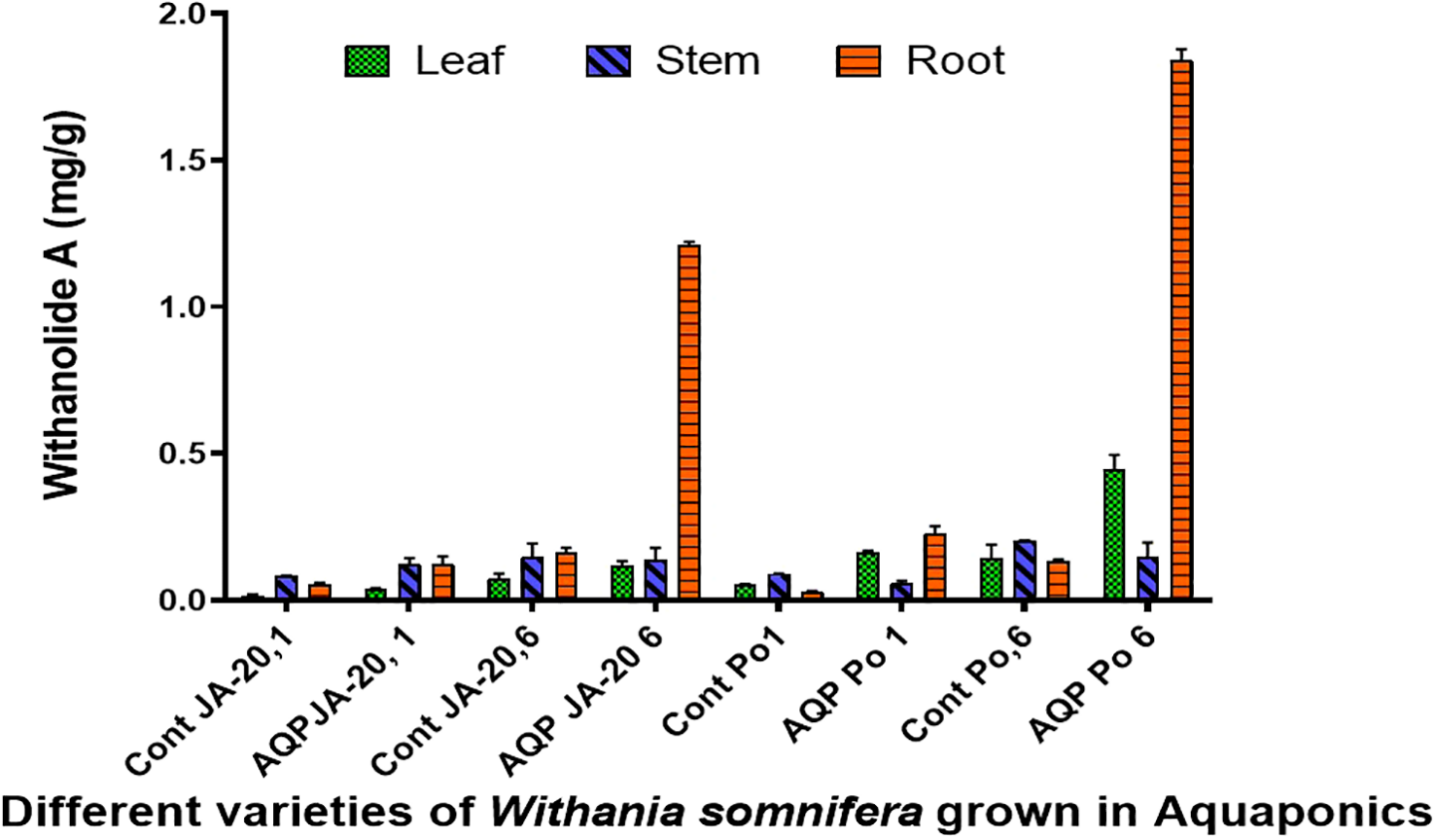
Withanolide A contents in Jawahar-20 and Poshita varieties of *Withania somnifera grown* in aquaponics (where Cont JA-20, 1 = 1 month Jawahar seedling grown in soil; AQP JA-20,1 = 1 month Jawahar seedling grown in aquaponics; Cont JA-20,6 = 6 month Jawahar seedling grown in soil; AQP JA-20,6 = 6 month Jawahar seedling grown in aquaponics; Cont PO1 = 1 month Poshita seedling grown in soil; AQP PO1 = 1 month Poshita seedling grown in aquaponics; Cont PO,6 = 6 month Poshita seedling grown in soil; AQP PO,6 = 6 month Poshita seedling grown in aquaponics).

The higher content of withaferin A was examined in the stem (1.407 mg/g), followed by the leaves (1.166 mg/g), and the root (0.331 mg/g) of Jawahar-20 in six-month-old plants. While the higher content of withanolide A was examined in the root (1.221 mg/g), followed by stem (0.177 mg/g), and leaves (0.133 mg/g) of Jawahar-20 in one-month-old plants. The six-month-old plants of Poshita showed a higher content of withaferin A in the stem (1.977 mg/g), followed by the leaves (1.499 mg/g), and the root (0.543 mg/g). While the six-month-old plants of Poshita showed a higher content of withanolide A in the root (1.879 mg/g), followed by the leaves (0.495 mg/g), and the stem (0.196 mg/g) (**Figure 4**). The summary of results is demonstrated in the **Supplementary Table. S1**. Hence, the aquafarming technique is an integrated farming method of growing plants under controlled environmental conditions. It is concluded that the stem and root have excellent contents of withaferin A and withanolide A antioxidant in the Jawahar-20 and Poshita varieties. Previous research on cherry tomatoes showed that the hydroponic system is very helpful in increasing the yield of crops by providing the necessary nutrients (Schmautz et al., 2016). Another published work also suggested the positive role of the microbial niche in the production of cherry tomatoes in the hydroponic system (Schmautz et al., 2017 *).*

### Analysis of essential elements and other parameters in the water sample of aquaponic culture

Various essential elements and other parameters were analyzed in the water sample of the aquaponics system and tap water used to water potted plants as control (**Table 1**).

The pH value was 6.5 in the aquaponic water sample and 7.7 in the control sample. The plants require a lower pH (5.5 to 6.5) for excellent growth in a hydroponic system (Rakocy et al., 2007). The results showed a high content of ammonia in the aquaponic water sample (i.e., 0.590 mg/l) with respect to the control sample (i.e., 0.253 mg/l). The content of Cu and Zn was found to be 0.308 μg/ml and 0.035 μg/ml in the water sample of the aquaponics system, respectively. The BOD was analysed at 2.95 mg/l in the water sample of the aquaponics system and 5.95 mg/l in the control sample, which provides a good condition for the aquaponics system (**Table 2**). The important role of BOD has been investigated in various studies, which illustrate that excess solid wastes are responsible for increasing BOD. The increase in BOD has promoted hypoxic conditions in the rhizosphere and may generate toxic concentrations of ammonia and nitrate (Rakocy et al., 2012; Danaher et al., 2013).

**Table 2:** Effect of aquaponics on plant biomass and yield of withaferin A and withanolide A in WS.

Aquaponics					
Treatments	Plant Height (cm)	Plant Biomass FW (g)	Plant Biomass DW (g)	Yield (Withaferin A) mg/Plant	Yield (Withanolide A) mg/Plant
Cont JA-20, 1	7.5^a^	1.2 ± 0.2^ab^	0.05 ± 0.01^ab^	0.23^a^	0.03^a^
AQP JA-20,1	18^b^	3.2 ± 0.5^bc^	0.1 ± 0.02^cb^	0.21^b^	0.01^e^
Cont JA-20,6	20^b^	2.9± 0.03^ab^	0.18 ± 0.01^bc^	0.2^f^	0.02^f^
AQP JA-20,6	25^c^	3.3 ± 0.2^bc^	0.2 ± 0.03^cd^	0.28^d^	0.03^e^
Cont PO1	9^a^	2.5 ± 0.28^cd^	0.04 ± 0.01^cd^	0.09^e^	0.03^a^
AQP PO1	20^b^	3.1± 0.2^ab^	0.0 ± 0.03^ab^	0.05^a^	0.01^a^
Cont PO,6	21^c^	3.2 ± 0.5^cd^	0.25± 0.02^ab^	0.28^c^	0.08^c^
AQP PO,6	32^d^	3.5 ± 0.04^ab^	0.3 ± 0.01^cd^	0.3^b^	0.09^b^

(Cont JA-20, 1 = 1 month Jawahar seedling grown in soil; AQP JA-20,1 = 1 month Jawahar seedling grown in aquaponics; Cont JA-20,6 = 6 month Jawahar seedling grown in soil; AQP JA-20,6 = 6 month Jawahar seedling grown in aquaponics; Cont PO1 = 1 month Poshita seedling grown in soil; AQP PO1 = 1 month Poshita seedling grown in aquaponics; Cont PO,6 = 6 month Poshita seedling grown in soil; AQP PO,6 = 6 month Poshita seedling grown in aquaponics).

Data shown are mean _ SEm (n = 3). The genotypes with same superscript within each assay (parameter) are not significantly different at p _ 0.05, according to Duncan multiple comparison procedure (ANOVA).

The micro/macro elements and physio-chemical properties play important roles in the growth of plants and the content of bioactive components (photochemical, antioxidant metabolites, proteins, enzymes, etc.) in aquaponics systems. The published work showed that among the different nitrogen sources tested, 15 mg/l ammonium nitrate considerably improved the levels of withanolides (1.74 mg/g DW withanolide A, 0.92 mg/g DW withanolide B, 0.52 mg/g DW withaferin A, and 1.54 mg/g DW withanone) in the roots of regenerated shoots after 4 weeks of culture (Sivanandhan et al., 2015). It has also been reported that aquaponics systems that solely rely on fish excreta have lower concentrations of phosphorous, potassium, iron, manganese, and sulphur, resulting in poor plant growth (Graber and Junge, 2009). So from the above data, it may be concluded that Poshita has a higher content of withaferin A and withanolide A as compared to Jawahar-20. The content of withaferin A and withanolide A was found to be highest in 6-month-old seedlings as compared to one-month-olds, which confirms that the content of withaferin A and withanolide A increases with age.

### Analysis of essential elements and other parameters in the soil sample

The results of various essential elements and other parameters were demonstrated in the soil samples of the mango garden, transgenic laboratory, and control sample using different techniques (**Table 3**).

**Table 3:** Element analysis of the soil sample used in the elicitation study for potting the plants in the mango garden and transgenic polyhouse.

S. No.	Parameters	Control	Mango Garden	Transgenic Laboratory
1.	Salinity(PSU)	33	44	94
2.	Electrical conductivity(µS/cm)	65	56	187
3.	pH	7.23	7.40	7.86
4.	Temperature(°C)	29	28.5	25
5.	Organic Carbon	12	6.3	4.3
6.	Potassium(K)	18.3	22.6	69.9mg/g
7.	Phosphorous	0.04 mg/g	0.035 mg/g	0.032 mg/g
8.	Nitrogen(N_2_)	3.3	3.8	4.2
9.	Vanadium	112.35 μg/g	117.96 μg/g	121.96 μg/g
10.	Chromium	79.06 μg/g	65.03 μg/g	69.50 μg/g
11.	Manganese	452.56 μg/g	402 μg/g	418.03 μg/g
12.	Iron	52063.91 μg/g	50569.36 μg/g	45235.25 μg/g
13.	Cobalt	62.36 μg/g	36.25 μg/g	30.24 μg/g
14.	Nickel	7.45 μg/g	6.98 μg/g	56.32 μg/g
15.	Copper	8.65 μg/g	75.25 μg/g	60.24 μg/g
16.	Zinc	39.57 μg/g	48.27 μg/g	52.45 μg/g

Both the growth of the plant and the content of withaferin A and withanolides are enhanced by the use of vermicompost as a biofertilizer. Vermicompost is a good source of plant nutrients, which may be very sustainable for crop production (Wani, 2002). Previous studies have reported that the fresh weight and dry matter of cowpea (*Vigna unguiculata*) were high when the soil was amended with vermicompost (Karmegan et al., 1999; Karmegam and Daniel, 2000). A similar positive response was obtained in sorghum (*Sorghum bicolor*) (Patil and Sheelavantar, 2000) and sunflower (*Helianthus annus*) (Devi and Agrawal, 1998). Kaur et al. (2018) have reported organic cultivation of ashwagandha with increased biomass and higher quantities of bioactive withanolides by using vermicompost. As the usage of synthetic fertilizer for improving crop production increases the cultivation cost and causes long-term harm to the biological ecosystem (Savci, 2012), it also results in the acidification of soil, reducing nutrient uptake (Campbell et al., 2008; Coolon et al., 2013).

## Conclusions

It is concluded that the Poshita variety has a higher content of withaferin A and withanolide A compared to Jawahar-20. The content of withaferin A and withanolide A was also found to increase with age. However, the major bottleneck is the availability of quality planting material/elite germplasm for enhanced active ingredients. The conventional propagation method cannot meet the increasing demand for this plant as a raw material for the preparation of pharmaceutical products or herbal formulations. Since environmental factors influence the secondary metabolite and antioxidant biosynthesis, it is therefore important to assess the withanolide A contents of elite germplasm and compare the withanolide A contents of field-grown and *in vitro-*grown promising varieties. The tissue culture technique can be an alternative for the continuous production of plantlet stocks for large-scale field cultivation and shoot multiplication. The most outstanding advantage offered by the aseptic mass propagation technique over conventional methods is that a large number of plants can be produced from a single plant. Unlike conventional methods of plant propagation, micropropagation of even temperature-specific species may be carried out throughout the year without any agro-climatic barriers. In order to enhance the contents of bioactive compounds, certain other strategies have been adopted by the researchers, like *Agrobacterium rhizogenes*-mediated transformation to increase the root biomass, the use of vermicompost, elicitors, suspension cultures, bioaugmented soil, etc. Looking into the facts and importance of *W. somnifera* as a medicinal herb that is being continuously depleted from its natural habitat in India, the present research aims to develop elite germplasm with higher withaferin A and withanolide A contents derived from the promising genotypes. Moreover, the developed germplasm should be maintained. The hardening and acclimatization of this germplasm is an important effort to develop *in vitro* accession lines so that it can be made available to farmers in fulfilment of commercial demands. The present study demonstrates that aquaponics can work as an essential driver for the development of integrated crop and food production systems. The dry regions, such as Saudi Arabia and the desert regions of India, suffering from scarcity of water will specifically gain huge benefits from aquaponics methods being started in the commercial environment.

## Data availability statement

The original contributions presented in the study are included in the article/**Supplementary Material**. Further inquiries can be directed to the corresponding author.

## Author contributions

MS, SB, ND: conducted the experiments, manuscript preparation. MS, KP, AA, MK, SCB: data analysis and editing. MS, AM, DCS: review, finalization of the manuscript and correspondence. All authors contributed to the article and approved the submitted version.
