# Supplementary material for: Estimating the production of withaferin A and withanolide A in Withania somnifera (L.) dunal using aquaponics for sustainable development in hill agriculture
Source: Front Plant Sci. 2023 Aug 31;14:1215592. doi: 10.3389/fpls.2023.1215592 (PMC10501395; doi:10.3389/fpls.2023.1215592)
Supplement: Supplementary file 1 [file DataSheet_1.doc]

**Supplementary Material**

**Methodology**

The samples of fresh leaves, stem and roots were taken from the one and six month old seedlings of Jawahar-20 and Poshita varieties of *Withania somnifera*. The tissue samples were subjected to drying in hot air oven at 40°C for 3-4 days until a constant dry weight was obtained. Then the plant tissue was grinded with the help of clean and dry mortar and pestle. 1g powder (dry weight) of plant tissue was taken and percolated in 50 ml 80% methanol, then sonicated for 20 minutes and placed on a rotatory shaker at 30°C at 100 rpm overnight. The procedure was repeated thrice and the methanolic extracts thus obtained after percolation were pooled together and filtered through Whatmann filter paper. Then the methanolic extract was subjected to drying using rotatory vacuum evaporator maintained at 60°C until completely dried residue was obtained .The dried residue was re-dissolved in HPLC grade methanol (4ml). A pinch of charcoal was added to the extract, in order to decolourise the sample and centrifuged at 8000 rpm for 15 min. The supernatant was then filtered through nylon filter membranes (0.22µ). The samples were used for the estimation of the content of Withaferin A and Withanolide A. The extra samples were kept in the vials at 4˚C for future uses **(Singh et al., 2018 and 2020).** The quantification of antioxidants was done through HPLC in one month old seedlings and six month old seedlings of Jawahar-20 and Poshita varieties of *Withania somnifera* (Singh et al., 2018).

**Essential elements and other parameters analysis in the water sample of aquaponic culture**

Analysis of elements present in the aquaponics water sample used for the elicitation experiment was done through the protocol of *Standard Methods for the examination of water and wastewater* by *American Public Health Association* (APHA) with few modifications.

**Atomic Absorption Spectrophotometer (AAS)**

The presence of Cadmium, Zinc, Copper, Chromium, and Lead was done by AAS. Digestion of sample (50 ml) with HNO_3_ (20 ml). Then the volume of the sample was reduced at a hot plate to 15 ml at 100°C. The final volume was made up to 100 ml with double distilled water. The sample was then filtered with Whatman filter paper and the reading of the sample was taken through AAS.

**Spectrophotometric Analysis**

The presence of Nitrite (NO_2_^-^), Ammonia (NH_3_^+^), and Phosphate (PO_4_^-^) was done through a spectrophotometer.

**Flame photometer**

The 5ml of the sample was taken and filtered with Whatman filter paper for the analysis of the presence of Sodium (Na) and Potassium (K). The equipment was calibrated with the standard solution of Na and K with a capillary tube (10, 20, 30, 40, 50, 60, 70,80,90 and 100 ppm). The sample was put in the equipment to take readings after calibration.

**Biological O_2_ Demand (BOD)**

BOD is the measurement of the amount of dissolved oxygen needed by aerobic biological organisms to break down the organic material present in a given water sample at a certain temperature over a certain time period.

**Neutralization of Sample**

50 ml of sample was taken in a 100 ml beaker. The pH of the solution was kept 7.0 by using 1N H_2_SO_4_ or 1N NaOH. The volume of H_2_SO_4_ or NaOH used to adjust the pH of 50 ml sample to 7.00 was recorded. Then the volume of sulfuric acid or sodium hydroxide required to neutralize the 1000 ml sample was calculated.

**Removal of Chlorine Content**

Removal of chlorine from the water sample was done by adding sodium sulfite to the sample. 50 ml of water sample was taken to which 2.5 ml of acetic acid (50%) was added followed by 2.5 ml of (10% w/v) solution of potassium iodide. After some time 1 ml of starch indicator was added and titrated with 0.025 N sodium sulfite solution.

**Preparation of Alkali-Iodide-Azide Reagent**

500 gm of sodium hydroxide (NaOH) and 135 gm of sodium iodide (NaI) were dissolved in distilled water. And the final volume was made up to 1000 ml of distilled water. To which 10 gm of sodium azide was added.

**Preparation of Dilution Water**

5 liters of double distilled water were taken in a glass container and aerated with clean compressed air for 12 hours. It was then allowed to get stable for at least 6 hours at 20 °C. After that 5 ml of 27.5% (w/v) solution of calcium carbonate, 5 ml of 22.5 % (w/v) solution of magnesium sulfate, 5 ml of 0.15% (w/v) solution of ferric chloride, and 5 ml phosphate buffer solution was added. The solution was mixed well and allowed to stand for 2 hours.

**Procedure to Determine the Biological Oxygen Demand of Water**

Four 300 ml BOD bottles were taken. In two bottles 10 ml of sample was added and the remaining volume was filled with dilution water. The other two BOD bottles were only filled with dilution water for blank. The bottles were closed immediately to avoid any air bubbles in the bottle. Then the bottles were incubated at 20 °C for 5 days. After 5 days the BOD of the sample was analyzed.

**Chemical O_2_ Demand (COD)**

Standards are prepared using KHP (potassium hydrogen phthalate). 2 ml of liquid was added to each vial. In the case of the “blank,” 2 ml of double distilled water was added. Then 2 ml of the standard was added to the corresponding vials. Each vial was mixed well and placed into the COD reactor block for two hours. After two hours, the vials were removed from the block to a cooling rack for about 15 minutes. The readings were taken with the help of a colorimeter.

**Analysis of essential elements and other parameters in the soil sample**

Various essential elements and other parameters were analyzed in the soil sample of the Mango garden, transgenic laboratory, and control soil sample. The soil (vermin-compost added and autoclaved) used for potting the plants of *Withania somnifera* in the Mango garden, Dept. of Plant Physiology, G.B Pant University of Agriculture and Technology and the other in the controlled environment of Transgenic Laboratory, Dept. Of Molecular Biology and Genetic Engineering, G.B Pant University of Agriculture and Technology was done through DTPA (Diethylene triamine penta acetic acid).

**Estimation of micronutrients in soil by extraction method**

10 g of air-dried soil was weighed in a 150 ml conical flask to which 20 ml of DTPA extraction buffer was added (1.967 g DTPA, 14.9 g TEA (Triethanolamine) and 1.47 g CaCl_2_.2H_2_O was dissolved in 200 ml DW). The pH of the solution was adjusted to 7.0 and the final volume was made up to 1000 ml. The flask was tightly capped with a polyethylene stopper and was then kept on a horizontal shaker (120 cycles/min) for 2 hrs. After shaking the filtrate was filtered through Whatman filter paper No. 42. and the filtrate was used for the estimation of micronutrients. The calculation of the extractable micro-nutrient content was done with the help of the following equation:

DTPA extractable micro-nutrient content (mg/kg soil) = filtrate concentration (µg/ml) x 20/10

**Table 1: Withaferin A and Withanolide A contents in the two varieties of *Withania somnifera* ( Jawahar-20 and Poshita)** grown in aquaponics

| **Jawahar-20** | | | | | | | | **Poshita** | | | | | |
| --- | --- | --- | --- | --- | --- | --- | --- | --- | --- | --- | --- | --- | --- |
| **Plant tissue** | | | **Withaferin A(mg/g DW)** | | **Withanolide A(mg/g DW)** | | | **Withaferin A(mg/g DW)** | | | **Withanolide A(mg/g DW)** | | |
| **Control (1 month)** | | | | | | | | | | | | | |
| Leaf | 0.111 ± 0.02^a^ | | | | 0.02 ± 0.02^a^ | | | 0.213± 0.0^b^ | | | 0.056 ± 0.01^a^ | | |
| Stem | 0.091 ± 0.002^a^ | | | | 0.084 ± 0.02^d^ | | | 0.077 ± 0.001^a^ | | | 0.091 ± 0.009^c^ | | |
| Root | 0.103 ± 0.01^a^ | | | | 0.059 ± 0.003^c^ | | | 0.103 ± 0.004^a^ | | | 0.031 ± 0.0^a^ | | |
| **Aquaponic seedling (1 month)** | | | | | | | | | | | | | |
| Leaf | 0.336 ± 0.03^b^ | | | | 0.041 ± 0.01^b^ | | | 0.400 ± 0.005^b^ | | | 0.168 ± 0.01^e^ | | |
| Stem | 0.446 ± 0.05^e^ | | | | 0.143 ± 0.0^f^ | | | 0.516 ± 0.01^a^ | | | 0.066 ± 0.001^b^ | | |
| Root | 0.367 ± 0.0^c^ | | | | 0.149 ± 0.0^f^ | | | 0.175 ± 0.0^a^ | | | 0.252 ± 0.01^g^ | | |
| **Control (6 month)** | | | | | | | | | | | | | |
| Leaf | | 0.498±0.01^f^ | | | | 0.09±0.002^d^ | | | 0.564±0.004^c^ | | | 0.189±0.009^ef^ | |
| Stem | | 0.398±0.03^d^ | | | | 0.193±0.08^h^ | | | 0.231±0.1^cd^ | | | 0.204±0.01^f^ | |
| Root | | 0.39±0.004^c^ | | | | 0.178±0.10^g^ | | | 0.115±0.009^b^ | | | 0.138±0.008^d^ | |
| **Aquaponic seedling (6 months)** | | | | | | | | | | | | | |
| **Jawahar-20** | | | | | | | | **Poshita** | | | | | |
| Leaf | 1.166 ± 0.02^g^ | | | | 0.133 ± 0.002^e^ | | | 1.499 ± 0.0^e^ | | | | 0.495 ± 0.001^h^ | |
| Stem | 1.407 ± 0.002^g^ | | | | 0.177 ± 0.005^g^ | | | 1.977 ± 0.0^f^ | | | | 0.196 ± 0.01^ef^ | |
| Root | 0.331 ± 0.01^b^ | | | | 1.221 ± 0.0^i^ | | | 0.543 ± 0.002^d^ | | | | 1.879 ± 0.003^i^ | |
|  | CD at 1% | | | CD at 5 % | CD at 1% | | CD at 5 % | CD at 1% | | CD at 5 % | | CD at 1% | CD at 5 % |
| Explants (A) | 0.004 | | | 0.003 | 0.004 | | 0.002 | 0.03 | | 0.02 | | 0.04 | 0.002 |
| Time in months (B) | 0.005 | | | 0.006 | 0.005 | | 0.004 | 0.04 | | 0.03 | | 0.03 | 0.004 |
| A*B | 0.008 | | | 0.009 | 0.009 | | 0.007 | 0.07 | | 0.05 | | 0.09 | 0.008 |
| CV | 4.5 | | | | 4.8 | | | 4.6 | | | | 4.2 | |

* **Data shown are mean±SE_m_ (n=3).**The genotypes with same superscript, within each assay (parameter) are not significantly different at p≤ 0.05, according to Duncan multiple comparison procedure.(ANOVA).
